# Chagas disease reactivation in a heart transplant patient infected by domestic *Trypanosoma cruzi* discrete typing unit I (TcI_DOM_)

**DOI:** 10.1186/s13071-015-1039-3

**Published:** 2015-08-25

**Authors:** Jaime A. Costales, Camille N. Kotton, Andrea C. Zurita-Leal, Josselyn Garcia-Perez, Martin S. Llewellyn, Louisa A. Messenger, Tapan Bhattacharyya, Barbara A. Burleigh

**Affiliations:** Centro de Investigación en Enfermedades Infecciosas, Escuela de Biología, Pontificia Universidad Católica del Ecuador, Avenida 12 de Octubre y Roca, Quito, Ecuador; Division of Infectious Diseases, Massachusetts General Hospital, Boston, USA; Present address: Wellcome Trust Centre for Molecular Parasitology, Institute of Infection and Inflammation, College of Medical, Veterinary and Life Science, University of Glasgow, Glasgow, UK; Present address: VIB Autoimmune Genetics Laboratory, Department of Microbiology and Immunology, KU Leuven, Leuven, Belgium; Department of Pathogen Molecular Biology, London School of Hygiene and Tropical Medicine, London, UK; Present address: Molecular Ecology and Fisheries Genetics Laboratory, School of Biological Sciences, University of Wales, Bangor, Deiniol Road, Bangor, Gwynedd LL57 2UW UK; Department of Immunology and Infectious Diseases, Harvard School of Public Health, Boston, USA

**Keywords:** *Trypanosoma cruzi*, Lineage, Reactivation, Chagasic cardiomyopathy, Chagas disease

## Abstract

**Background:**

*Trypanosoma cruzi,* causative agent of Chagas disease, displays high intraspecific genetic diversity: six genetic lineages or discrete typing units (DTUs) are currently recognized, termed TcI through TcVI. Each DTU presents a particular distribution pattern across the Americas, and is loosely associated with different transmission cycles and hosts. Several DTUs are known to circulate in Central America. It has been previously suggested that TcI infection is benign and does not lead to chronic chagasic cardiomyopathy (CCC).

**Findings:**

In this study, we genotyped *T. cruzi* parasites circulating in the blood and from explanted cardiac tissue of an El Salvadorian patient who developed reactivation Chagas disease while on immunosuppressive medications after undergoing heart transplant in the U.S. as treatment for end-stage CCC. Parasite typing was performed through molecular methods (restriction fragment length polymorphism of polymerase reaction chain amplified products, microsatellite typing, maxicircle sequence typing and low-stringency single primer PCR, [LSSP-PCR]) as well as lineage-specific serology. We show that the parasites infecting the patient belong to the TcI DTU exclusively. Our data indicate that the parasites isolated from the patient belong to a genotype frequently associated with human infection throughout the Americas (TcI_DOM_).

**Conclusions:**

Our results constitute compelling evidence in support of TcI DTU’s ability to cause end-stage CCC and help dispel any residual bias that infection with this lineage is benign, pointing to the need for increased surveillance for dissemination of this genotype in endemic regions, the USA and globally.

## Findings

Chagas disease, caused by *Trypanosoma cruzi*, is the most important parasitic disease in Latin America, [1] and constitutes an emerging global public health problem, since thousands of *T. cruzi-* infected Latin Americans migrated during the last few decades and now live in North America, Europe, Australia, Japan and other regions [2]. A spectrum of clinical manifestations may result from human infection with *T. cruzi*, ranging from a total absence of symptoms to extremely debilitating and often deadly cardiac or digestive syndromes [3], and *T. cruzi*’s genetic diversity is suspected to influence the clinical outcome. Six major genetic lineages or discrete typing units (DTUs) are currently recognized (named TcI through TcVI), each displaying different biological characteristics [4]. There are as yet no proven associations between *T. cruzi* genetic lineages and the clinical presentations of the disease; DTUs TcII, V and VI are frequently reported to be present in the Southern Cone of South America where serious chronic manifestations include megaesophagus and megacolon, whereas TcI is reported to predominate in endemic countries north of the Amazon [4]. The pathogenicity of this lineage is evident in acute cases, where patent clinical manifestations, including death, have been reported in TcI oral outbreaks [5]. However, in terms of chronic symptoms, TcI has been suggested to be benign [6], with observed chronic chagasic cardiomyopathy (CCC) in TcI-infected patients attributed to co-infection with other *T. cruzi* DTUs.

Despite being based on limited sampling, this view has been reiterated in additional publications [7–9]. Furthermore, it has also been suggested that Chagas disease may be less severe among patients in the Amazon basin, where TcI infection is likely [10]. TcI exhibits high intra-lineage diversity, with members of a widely dispersed subgroup (termed TcI_DOM_) associated with many human infections [11], although no direct link with clinical manifestations has been established.

Here, we report a case of chronic chagasic cardiomyopathy and reactivation disease accompanied by extensive molecular characterization of the *T. cruzi* parasites involved. A 43 year old chagasic patient from El Salvador was admitted to Massachusetts General Hospital to undergo orthotropic heart transplantation. Immunofluorescence assay to detect anti-*T. cruzi* IgG previously performed at the Centers for Disease Control & Prevention (CDC, Atlanta, USA) was positive at >1:256 (diagnostic threshold > 1:32). His cardiac symptoms corresponded to New York Heart Association class IV and he had a biventricular pacemaker/defibrillator for complete heart block. Fifteen months previously, he underwent mitral valve replacement, tricuspid valve reconstruction and partial left ventriculectomy, at which time a left ventricle excision and atrial appendage biopsy showed active and healed myocarditis, consistent with late phase Chagas disease. He had no other known etiologies for his heart failure. Orthotropic heart transplantation was successful. Pathologic examination of the explanted heart revealed findings consistent with end-stage CCC, including dilatation with near complete atrophy of the left ventricular wall, endocardial fibrosis, diffuse myocardial fibrosis, and mononuclear infiltrates with some eosinophils and neutrophils. The infiltrate (lymphocytic myocarditis) was composed of lymphocytes (many CD3 T cells, more CD8 than CD4, few CD20 B cells) plus many CD68 macrophages. Amastigotes were not identified on multiple sections examined; however, *T. cruzi* kinetoplast DNA was detected by PCR in frozen tissue submitted to the Parasitic Diseases and Diagnostics Branch of the CDC. Weekly microscopic examination of his blood was performed after transplant, screening for early detection of reactivation disease; this was positive with rare trypomastigotes detected at week six after transplant. He was given nifurtimox 8-10 mg/kg (as 120 mg four times a day) orally for 10 weeks, and developed severe peripheral neuropathy with anorexia. He was switched to benznidazole, 150 mg twice a day for 30 days, which he tolerated well, and his peripheral neuropathy and anorexia resolved. He was monitored monthly for parasitemia for approximately one year after the end of therapy, with no evidence of further infection.

**Fig. 1:**
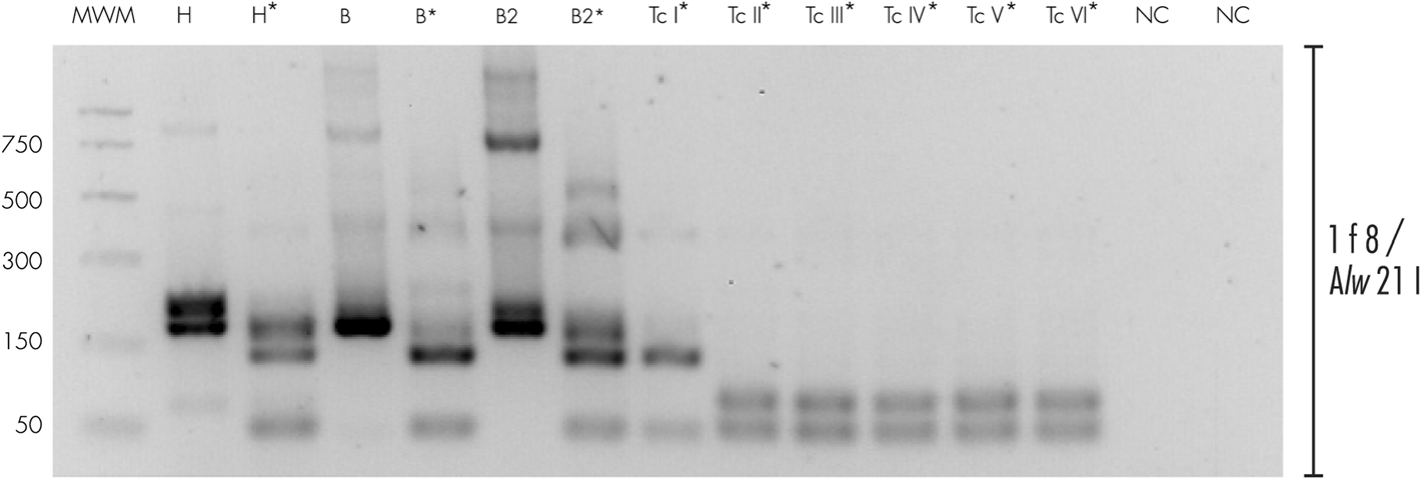
Molecular typing from clinical samples. DNA was extracted from blood samples and parafinized heart explants. Typing was performed using a nested PCR-RFLP strategy for amplification of the 1f8 flagellar protein and digestion with *Alw* 21I (Van der Auwera, et al., unpublished data). H = DNA extracted from parafinized heart explant tissue, B and B2 = blood samples taken 10 days apart during reactivation of disease, TcI-TcVI correspond to DTU controls, NC lanes correspond to negative controls for the PCR and nested-PCR. Lanes corresponding to *Alw* 21I restriction digest products are labeled with an asterisk (*). Only restriction products are shown for controls

**Fig. 2:**
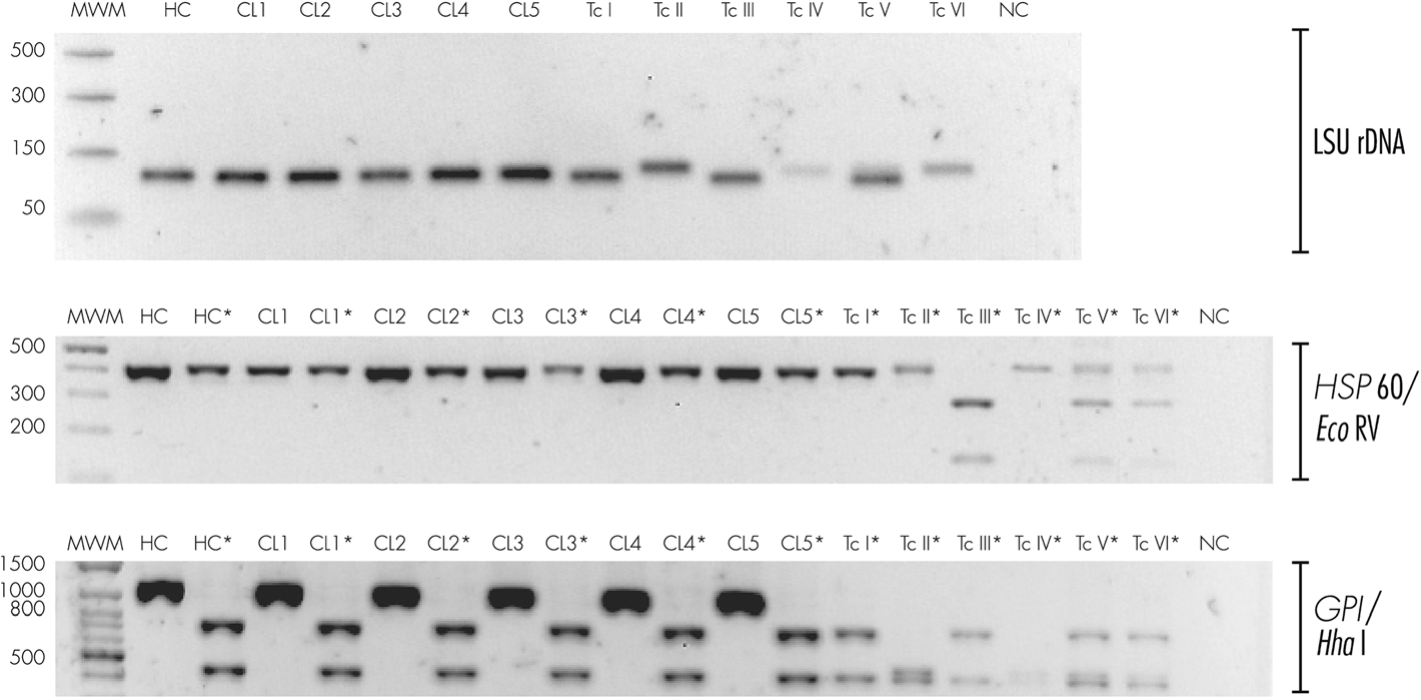
Molecular typing for cultured parasites and parasite clones. DNA was extracted from epimastigote hemocultures (HC) and five derived clones (CL1-CL5). TcI-TcVI correspond to DTU controls, NC corresponds PCR negative control. Lanes containing restriction products are labeled with an asterisk (*). Only restriction products are shown for controls. DNA was analyzed by the PCR-RFLP scheme proposed by Lewis, et al., 2009 [12]: as indicated by the brackets on the right side, fragments from the LSUrDNA, *HSP*60 and *GPI* genes were amplified by PCR. *GPI* and *HSP*60 products were digested with *Hha*I and *Eco*RV restriction enzimes, respectively

**Fig. 3:**
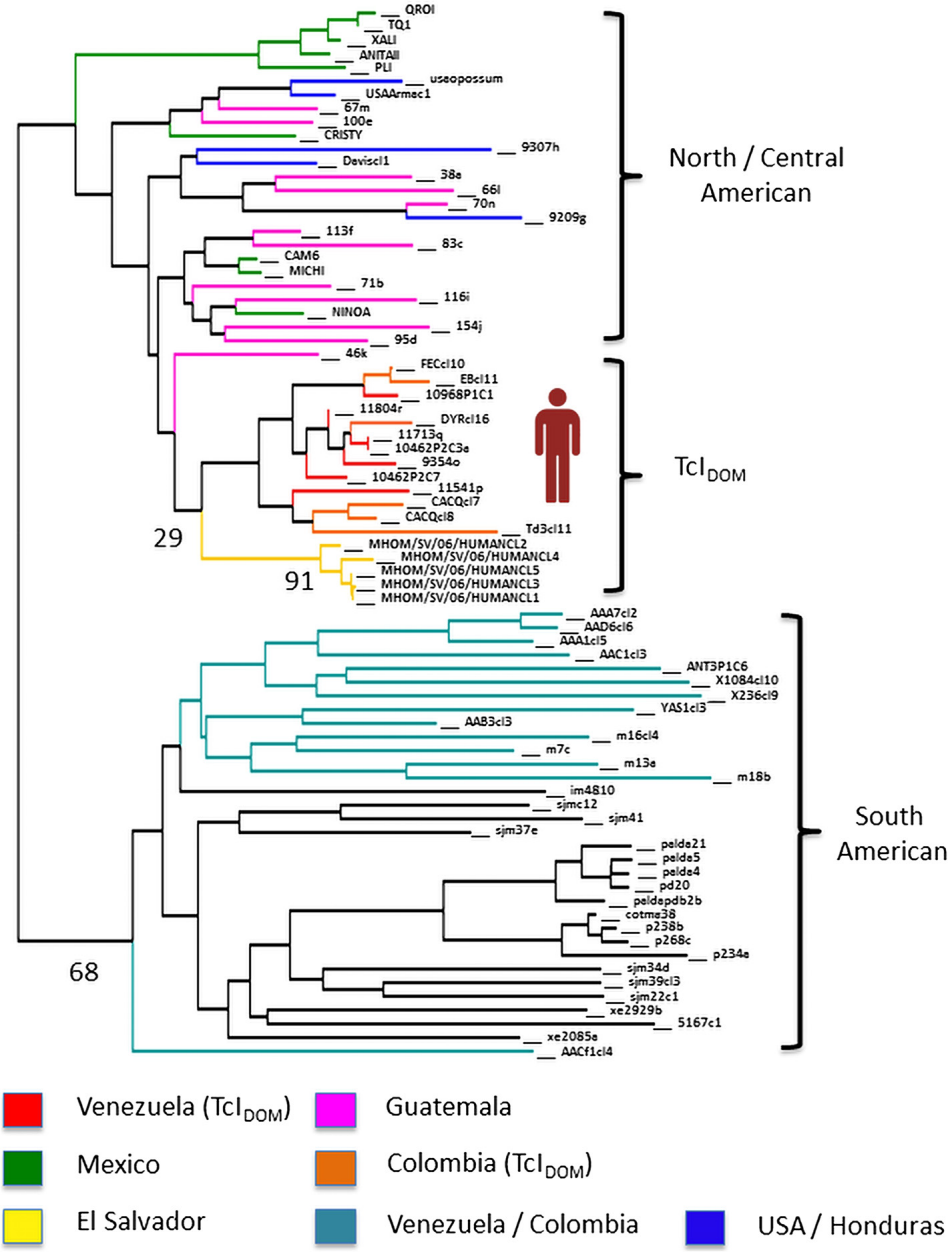
Neighbour joining dendrogram based on pairwise inverse allele sharing. Relationship between parasite clones isolated from the patient and others from North, Central and South America are shown. Branch colours indicate strain origin and values at important nodes indicate percentage of bootstrap support over 1000 trees. Further details of strains and analytical strategy can be found in [11]

**Fig. 4:**
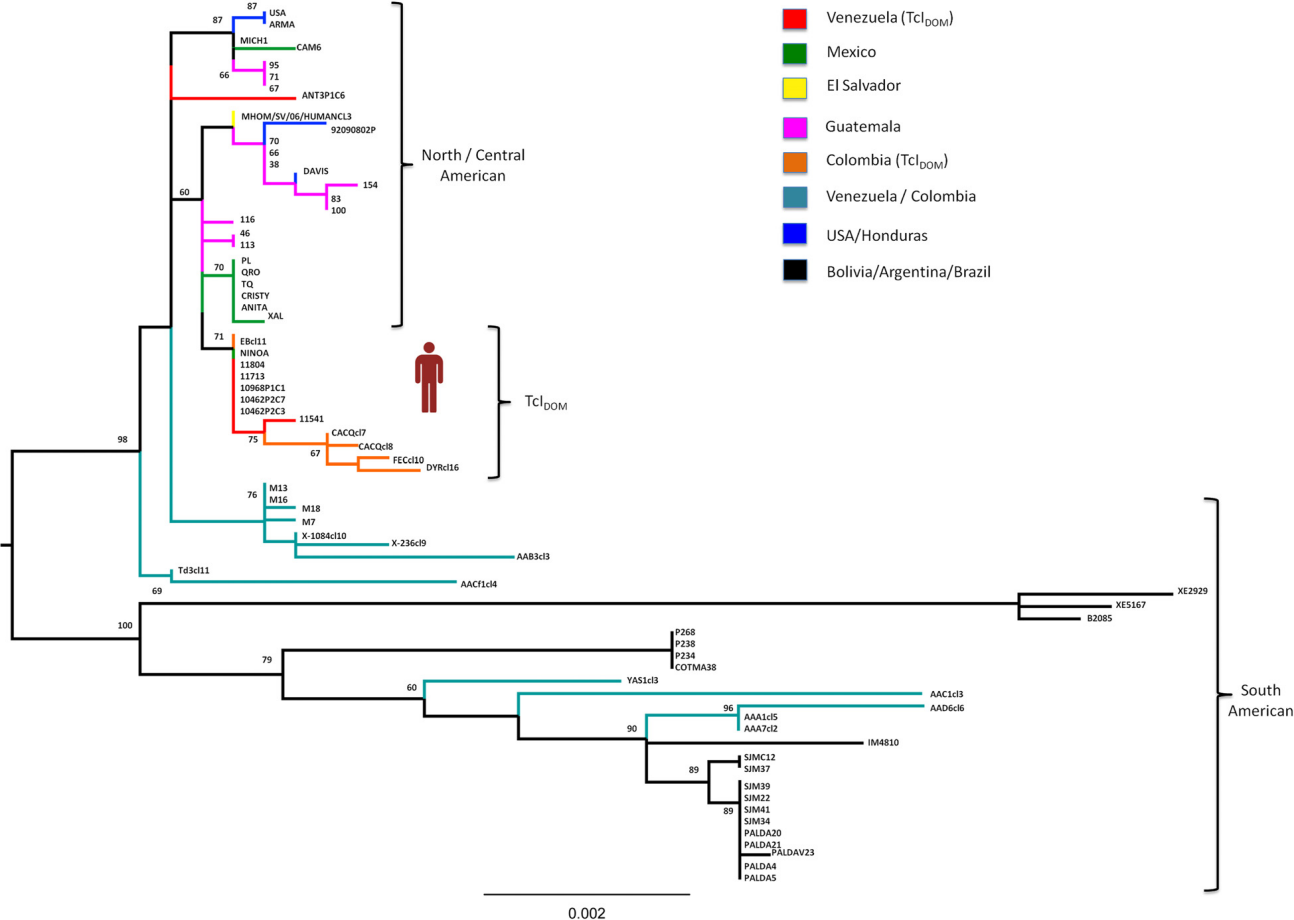
Maxicircle sequence-based typing of strain isolated from patient. Maxicircle sequences for one biological clone were concatenated (accession # KP136828) according to [14], aligned against 70 TcI strains encompassing TcI genetic diversity from across North, Central and South America [11] and used to assemble a Maximum-Likelihood topology in PhyML. The best-fit model of nucleotide substitution was selected from 88 models and its significance evaluated according to the Akaike Information Criterion (AIC) in jMODELTEST 1.0. The best fit model selected for this dataset was GTR + G. Bootstrap support for clade topologies was estimated following the generation of 100 pseudo-replicate datasets

**Fig. 5:**
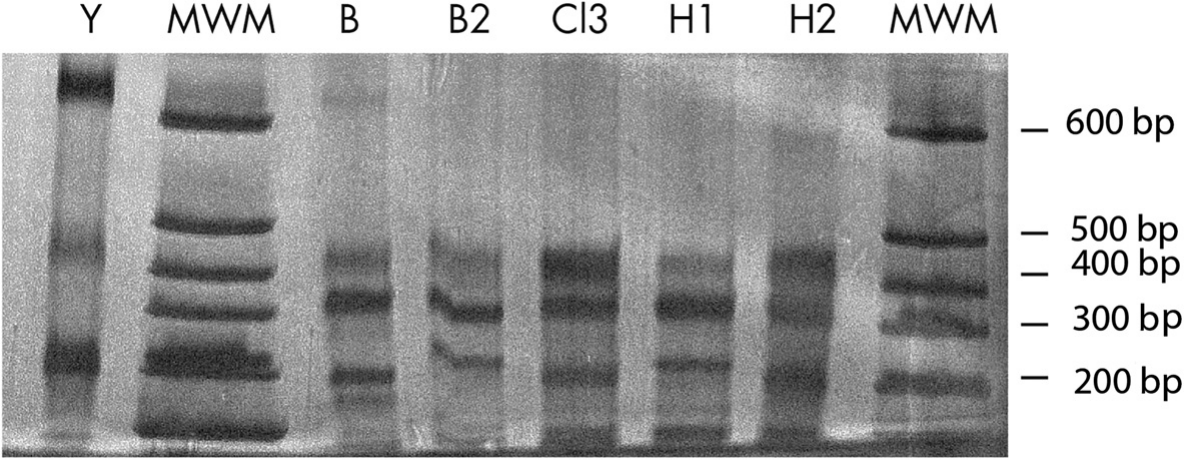
LSSP-PCR. DNA from parafinized heart explants and blood samples was amplified using the S35/S36 primer pair. The 330 bp product was gel-purified and analyzed by LSSP-PCR. Products were run in a 10 % polyacrilamide gel and silver-stained. H1 and H2 correspond to DNA extracted from two different sections of parafinized heart explant tissue B and B2 correspond to blood samples taken 10 days apart, starting six weeks after transplant. Cl3 corresponds to DNA from one of the clones derived from the original *T. cruzi*isolate. Y indicates “Y strain” parasites (TcII DTU) used for comparison. MWM = molecular weight markers

Genotyping directly from patient’s blood samples and parafinized heart explants using a nested PCR-RFLP for the 1f8 flagellar protein and digestion with *Alw*21I restriction enzyme (Van der Auwera, unpublished) assigned parasites to DTU TcI (Fig. 1). Hemoculture six and eight weeks after transplant yielded epimastigotes, which were cloned in solid medium. Cultured parasites and clones were determined to belong to DTU TcI by polymerase chain reaction and restriction fragment length polymorphism (PCR-RFLP) as in [12] (Fig. 2). Intra-TcI genotyping was performed with nuclear microsatellites [13] and maxicircle gene fragments [14]. Intriguingly, microsatellite data indicate a close relationship with TcI_DOM_, a distinct genotype within DTU TcI which is common among human cases in Latin America (Fig. 3), while the maxicircle sequence analysis indicates an origin among wild/non-human isolates for North and Central America (Fig. 4). Additionally, LSSP-PCR [15] showed matching patterns among DNA extracted from patient blood samples, heart explants and cultured parasite clones, indicating that they represent the same parasite population. Meanwhile, Y-strain, from the TcII lineage, employed as a control, yielded a completely different amplification pattern (Fig. 5).

Finally, although patient’s serum reacted against *T. cruzi* antigen in three different commercial serological tests (Chagatek-Biomerieux, Chagas III-Abbott BiosChile and Chagatest Recombinante 3.0-Wiener Lab), it did not recognize synthetic peptides derived from the TSSA antigen specific for DTUs TcII, III, IV or V/VI described in [16]. Therefore, we conclude that the observed CCC was caused by TcI, unequivocally confirming that TcI is an agent of life threatening heart disease.

TcI constitutes the most abundant and widespread *T. cruzi* DTU [4] and is the predominant DTU in the Amazon region and countries North of it [1]. For our patient, only TcI DTU parasites were detectable in the clinical samples, hemocultures, and biologically cloned parasites from hemoculture. Furthermore, antibodies against TcII, III, IV or V/VI - specific peptide epitopes were not detectable in serum, suggesting the absence of co-infection or previous infection with those lineages; there is currently no adequately sensitive equivalent TcI-specific serological assay [16].

TcI has considerable intra-DTU diversity [13]; specific genotypes within TcI are associated with human infection [11]. Based on nuclear microsatellite information, the patient was infected by TcI_DOM,_ a genotype associated with many human infections in regions north of the Amazon. TcI_DOM_ has not previously been reported from cardiac tissues in CCC cases, as it has been merely detected in peripheral blood or hemoculture, where co-infection with parasites from other genetic lineages causing CCC cannot be ruled out. Remarkably, mitochondrial genotyping suggests a closer relationship with isolates from North and Central America, consistent with local, possibly sylvatic, origin of the infecting strain. Given the proclivity for mitochondrial introgression into TcI_DOM_ [11, 14, 17, 18] we suggest our observation represents yet another such hybrid among many, in this case between TcI_DOM_ and a local strain, highlighting the need for control strategies aimed at domiciliary and extrinsic parasite populations as sources of human infection.

The case portrayed herein highlights many of the challenges that may arise when encountering a case of CCC (end-stage heart failure, need for heart transplant, Chagas disease reactivation under immune suppression, adverse reaction to parasiticidal treatment) which were very competently handled by a capable medical team in a non-endemic nation. Awareness of Chagas disease as an emerging global problem is essential for successfully meeting such challenges, which are predicted to be faced with increased frequency in non-endemic countries.

Furthermore, the results of our molecular characterization constitute compelling evidence in support of the role of TcI as causative agent of end-stage CCC and help dispel any residual bias that infection with this lineage is benign. Considering the broad distribution of TcI (the only *T. cruzi* DTU ranging from the Southern United States to Argentina and Chile) and the frequency with which TcI strains are associated with human infection [4], there is need for greater surveillance in TcI endemic regions like Central America. Around 22 million people from Chagas endemic countries live in the US, and most of these immigrants come from Mexico (74 %) and El Salvador (6.4 %) [2], where TcI is known to predominate [4]. Thus, a significant proportion of the estimated 300,000 *T. cruzi* infections among immigrants in the U.S.A. are predicted to involve the DTU TcI, adding to the growing economic burden of medical care and interventions associated with Chagas disease in the U.S.A., including transplantation for end-stage heart disease.

### Ethical approval

The study was approved by MGH’s Internal Review Board and written informed consent was obtained from the patient.

## Abbreviations

CCC: Chronic chagasic cardiomyopathy
DTU: Discrete typing unit
TcI: *Trypanosoma cruzi* lineage I
TcI_DOM_: Domiciliary population of *Trypanosoma cruzi* lineage I frequently associated with human infection
LSSP-PCR: Low stringency single primer polymerase chain reaction
